# Linguistic Influences on Cognitive Test Performance: Examinee Characteristics Are More Important than Test Characteristics

**DOI:** 10.3390/jintelligence10010008

**Published:** 2022-01-27

**Authors:** Damien C. Cormier, Okan Bulut, Kevin S. McGrew, Kathleen Kennedy

**Affiliations:** 1 Department of Educational Psychology, University of Alberta, Edmonton, AB T6G 2G5, Canada; kk4@ualberta.ca; 2 Centre for Research in Applied Measurement and Evaluation, University of Alberta, Edmonton, AB T6G 2G5, Canada; 3 Institute for Community Integration, University of Minnesota and Institute for Applied Psychometrics, St. Joseph, MN 56374, USA; iqmcgrew@gmail.com

**Keywords:** psychoeducational assessment, cognitive abilities, language abilities, school psychology, clinical psychology

## Abstract

Consideration of the influence of English language skills during testing is an understandable requirement for fair and valid cognitive test interpretation. Several professional standards and expert recommendations exist to guide psychologists as they attempt to engage in best practices when assessing English learners (ELs). Nonetheless, relatively few evidence-based recommendations for practice have been specified for psychologists. To address this issue, we used a mixed-effects modeling approach to examine the influences of test characteristics (i.e., test directions) and examinee characteristics (i.e., expressive and receptive language abilities) on cognitive test performance. Our results suggest that language abilities appear to have a significant influence on cognitive test performance, whereas test characteristics do not influence performance, after accounting for language abilities. Implications for practice include the assessment of expressive and receptive language abilities of EL students prior to administering, scoring, and interpreting cognitive test scores.

## 1. Introduction

It is widely accepted that diagnostic decisions need to be based on strong assessment practices. There are several indicators suggesting that the diagnostic process can be challenging when a standardized, norm-referenced test is used to assess students from diverse backgrounds. One is the disproportionate number of students from diverse backgrounds qualifying for special education services under a variety of disability categories (Harry and Klingner 2014). This problem has been described as being “among the most longstanding and intransigent issues in the field” (Skiba et al. 2008, p. 264). Although multiple factors contribute to the diagnostic process, some researchers have focused their attention on a type of test (e.g., cognitive measures) used to inform their diagnostic decisions (e.g., Cormier et al. 2014; Styck and Watkins 2013). However, even as researchers focus on one type of test, there is a myriad of potential contributing factors that they examine. For example, some researchers have investigated potential differences in the linguistic environments of students from diverse backgrounds compared to those of the majority, which are better represented in a test’s normative sample (Ortiz et al. 2012). Regardless of their focus, one of the limitations of many of these studies is that the group of interest is defined as culturally and linguistically diverse students, which is very broad. More recently, Ortiz (2019) has argued for a narrower focus on English learners (EL), so recommendations for practice can be applied more appropriately by considering a single attribute, such as their language abilities.

English learner (EL) has frequently been used as the primary descriptor for the group of students who are likely going to struggle to demonstrate their true abilities on a standardized measure that is normed using an English-speaking population (Ortiz 2019). EL specifically refers to students who are non-native English speakers with acquired proficiency in a second language (Ortiz 2019). At times, the term EL is confused with bilingual. This confusion is problematic because the word bilingual does not necessarily imply that English is the second language acquired by the student. EL is more appropriate given that the students within this group can fall at various points along the English proficiency continuum (Ortiz 2019). Furthermore, any given level of English proficiency does not negate the fact that these students are either continuing to develop their proficiency skills in English or are working towards doing so (Ortiz 2019). To improve accuracy in diagnostic decisions, psychologists and other professionals conducting assessments must take a comprehensive approach to understand both the examinee and test characteristics that may influence test performance for EL students when they are administered standardized, norm-referenced tests.

### 1.1. Assessing the Abilities of EL Students

The valid assessment of EL students often poses a significant challenge for psychologists. Although a number of broad and specific professional standards exist, best practices are still unclear. For example, Standard 9.9 from the *Standards for Psychological and Educational Testing* (AERA et al. 2014) emphasizes the requirement of having a “sound rationale and empirical evidence, when possible, for concluding that […] the validity of interpretations based on the scores will not be compromised” (p. 144). The *Standards* further stress that when a test is administered to an EL student, “the test user should investigate the validity of the score interpretations for test-takers with limited proficiency in the language of the test” (p. 145). The commentary associated with this specific standard highlights the responsibility of the psychologist in ensuring that language proficiency is minimized as a factor in the interpretation of the test scores.

If practicing psychologists were to consult the available evidence, they would need to weigh multiple variables and attempt to infer the extent to which a particular student’s assessment data is influenced by such variables. To date, some studies have suggested varying degrees of observed score attenuation for linguistically diverse students (e.g., Cormier et al. 2014; Kranzler et al. 2010; Tychanska 2009). Other studies have reported that the rate of differentiation between groups is no better than chance (Styck and Watkins 2013). Regardless of the underlying causal variable, these studies only suggest that practitioners should be cautious when interpreting test results. Thus, the extant literature does not provide specific recommendations or strategies for generating or testing hypotheses that would help practitioners better understand which test or examinee characteristics influence observed test score performance. Nonetheless, the two key areas initially identified and described by Flanagan et al. (2007)—test characteristics and examinee characteristics—continue to be focal areas of interest, as researchers attempt to develop approaches to assessing EL students that are in line with best practices.

#### 1.1.1. Test Characteristics

For decades, the content, administration, scoring, and interpretation of tests—essentially, tests’ psychometric properties—were highly criticized as producing biased scores for EL students (see Reynolds 2000, for a comprehensive review). Years of research produced arguments (e.g., Helms 1992) and counterarguments (e.g., Brown et al. 1999; Jensen 1980) with respect to this controversy. Although there was some evidence of bias in the measures used in previous decades, test developers appear to have taken note of the limitations of previous editions and, as a result, contemporary measures have significantly reduced the psychometric biases between groups (Reynolds 2000).

Despite these advances within the test development process, researchers have continued to investigate potential issues. For example, Cormier et al. (2011) were the first to quantify the linguistic demand of the directions associated with the administration of a standardized measure. Their goal was to examine the relative influence of this variable across a measure’s individual tests (i.e., subtests); if there was considerable variability among a measure’s individual tests, then this may be a meaningful variable to consider as practitioners selected their battery when assessing EL students. They accomplished this goal by using an approach suggested by Dr. John “Jack” Carroll, a prominent scholar who was not only a major contributor to the initial Cattell-Horn-Carroll (CHC) Theory of Intelligence, but who also had a passion for psycholinguistics. The methodology involved using text readability formulas to approximate the linguistic demand of test directions required with the 20 tests from the Woodcock-Johnson Tests of Cognitive Abilities, Third Edition (WJ III; Woodcock et al. 2001). A subsequent study (Cormier et al. 2016) investigated the linguistic demand of test directions across two editions of the same cognitive battery—the WJ III and the Woodcock-Johnson Tests of Cognitive Abilities, Fourth Edition (WJ IV COG; Schrank et al. 2014a). Eventually, Cormier et al. (2018) identified several test outliers across commonly used cognitive test batteries by applying the methodology used by Cormier et al. (2011). Although this series of studies appears to have produced meaningful recommendations to practitioners with respect to test selection, the extent to which test directions have a significant influence on the actual performance of examinees remains unknown.

#### 1.1.2. Examinee Characteristics

Standardized measures are constructed based on the presumption that the students who are assessed using the measures possess a normative level of English proficiency. (Flanagan et al. 2007). Under this presumption, the average examinee would be able to understand test directions, produce verbal responses, or otherwise use their English language skills at a level that is consistent with their peers (Flanagan et al. 2007). However, as noted by Ortiz (2019), there is likely to be a continuum of language levels within an EL population. Unfortunately, researchers have not focused on the level of English language proficiency as a way of differentiating performance on standardized measures. As a result, much of the research completed to date only provides information on general group-level trends that may or may not be observed by practitioners *after* they have administered a battery of standardized measures. One of the potential reasons for the lack of consistency may be related to the population of interest being defined as culturally *and* linguistically diverse, instead of focusing on a specific, measurable student characteristic, such as English language proficiency. As a result, practitioners may be reluctant to incorporate additional measures into their assessment batteries when testing EL students because: (a) there is no clear definition of the type of student these recommendations would apply to; and (b) the evidence regarding the validity of patterns of performance on standardized measures is, at best, still mixed (e.g., Styck and Watkins 2013). Thus, there appears to be a need to examine the influence of both test and examinee characteristics together to better understand the impact of these characteristics on test performance when standardized measures are used.

### 1.2. Current Study

A review of the literature has led to the conclusion that researchers have, perhaps, overlooked the potential of considering examinee characteristics as they attempt to produce research with empirical recommendations for the assessment of EL students. Moreover, the data produced by Cormier et al. (2018) provide a quantification of the linguistic demands of many commonly used measures of cognitive abilities. Taken together, it is now possible to investigate test and examinee characteristics together, to understand their relative contributions to performance on standardized measures. Consequently, we sought to answer the following research question: What are the relative contributions of test characteristics (i.e., the linguistic demand of test directions) and examinee characteristics (i.e., oral English language skills) to performance on a standardized measure of cognitive abilities?

## 2. Materials and Methods

### 2.1. Sample

The normative sample for the Woodcock-Johnson IV (Schrank et al. 2014b) was the primary source of data for this study. The Woodcock-Johnson IV is a battery of 51 tests that includes the WJ IV COG, the Woodcock-Johnson Tests of Academic Achievement (WJ IV ACH; Schrank et al. 2014c), and the Woodcock-Johnson Tests of Oral Language (WJ IV OL; Schrank et al. 2014d). The stratified sampling design included variables such as region, sex, country of birth, race, ethnicity, community type, parent education, and educational attainment. For the purpose of this study, we used the school-age sub-sample, which resulted in a sample size of 4212 students.

### 2.2. Measures

The WJ IV COG is comprised of 18 individual tests; 10 for the standard battery and eight for the extended battery (see Table 1 for a list of the WJ IV COG tests). Four of the WJ IV COG tests—Oral Vocabulary, Phonological Processing, Visualization, and General Information—contain subtests (see Table 1). The 18 tests of cognitive abilities were developed for the purpose of “measuring general intellectual ability, broad and narrow cognitive abilities, academic domain-specific aptitudes, and related aspects of cognitive functioning” (McGrew et al. 2014, p. 8). Seven tests from the WJ IV COG are used to generate a General Intellectual Ability (GIA) score for assessing the higher-order psychometric *g* construct from the CHC Theory of Intelligence. Overall, the WJ IV COG demonstrates excellent technical adequacy (see Reynolds and Niileksela 2015 for a comprehensive review of the WJ IV COG). For this study, we used individual test and subtest scores, which also demonstrate strong psychometric properties. For example, the internal consistency coefficients for the WJ IV COG individual tests range “from 0.74 to 0.97, with a median reliability of 0.89” (Reynolds and Niileksela 2015, pp. 387–88). The validity evidence is also strong and was described as “strikingly comprehensive” (p. 388).

The WJ IV OL is comprised of 12 tests (see Table 1 for a list of the WJ IV OL tests). The stated purpose of the WJ IV OL is to measure “oral language ability and listening comprehension (in English or Spanish), oral expression, and two important cognitive-linguistic abilities: phonetic coding and speed of lexical access” (McGrew et al. 2014, p. 10). For this study, we used the Oral Expression and Listening Comprehension cluster scores that are administered in English only (see Table 1 for test details). The median reliability coefficients for the Oral Expression and Listening Comprehension clusters are 0.89 and 0.90, respectively (McGrew et al. 2014, p. 291).

As noted previously, the study completed by Cormier and colleagues produced quantitative values for the relative influence of the linguistic demand of test directions across cognitive assessment batteries. Their investigation included test directions from four norm-referenced measures of cognitive abilities: the Cognitive Assessment System, Second Edition, the Kaufman Assessment Battery for Children, Second Edition, the Wechsler Intelligence Scale for Children, Fifth Edition, and the WJ IV COG. A total of 99 individual tests and subtests from these four measures were included in their analyses. Principal component analyses produced values ranging from −0.96 to 5.37 for standard test directions and values ranging from −0.57 to 8.39 for supplementary test directions.

The relative linguistic demand values for the WJ IV COG used for analysis were taken from their results. The analysis was completed at the subtest level, when applicable because unique directions are provided for each subtest. The only exception is the General Information subtests, *Where* and *When*, which was represented at the test level in the analysis completed by Cormier and colleagues, presumably due to their data coding rules. Thus, the final tests and subtests from the WJ IV COG used for the purposes of this study are listed in Table 1.

### 2.3. Procedure

To determine the relative contributions of the variables of interest, we employed a mixed-effects modeling approach. Unlike linear regression models that can only estimate fixed effects for independent variables, mixed-effects models can incorporate both fixed and random effects within a multilevel structure. In the context of mixed-effects modeling, random effects refer to parameters of independent variables that represent a random sample of variables drawn from a population with mean μ and standard deviation σ. A typical mixed-effects model can be expressed in matrix form as follows:

(1) $$y_{i}=X_{i}\beta +Z_{i}b_{i}+\varepsilon_{i},$$

where $y_{i}$ is the vector of the dependent variable *y* for group *i* (*i* = 1, 2, 3, …, *K*) where individual observations (level 1) are nested within the group (level 2), $X_{i}$ is the matrix of the fixed-effect predictors for group *i*, β is the vector of fixed-effect coefficients which are the same for all groups, $Z_{i}$ is the matrix of the random effects for group *i*, $b_{i}$ is the vector of random-effect coefficients for group *i*, and $\varepsilon_{i}$ is the error (i.e., residual) term for individual observations in group *i*. In Equation (1), both $b_{i}$ and $\varepsilon_{i}$ are assumed to be normally distributed.

In this study, we assume a multilevel structure where students (level 1) are nested within the tests of the WJ IV COG (level 2). That is, the tests from the WJ IV COG represent a sample of tests drawn from the population of all possible cognitive tests. To analyze the effects of the student- and test-related predictors, we developed six mixed-effects models (i.e., Models 1 to 6). We opted to use *W* scale scores from the WJ IV COG and the WJ IV OL, which are “a direct transformation of the Rasch logit scale” (McGrew et al. 2014, p. 46). The *W* scores are centered on a value of 500, which eliminates the possibility of negative ability scores. Model 1 aimed to demonstrate the variation in students’ *W* scores across the tests, and thus it did not include any fixed-effect predictors. Model 2 included age and the GIA scores as fixed-effect predictors to account for the variation in the test scores due to students’ cognitive development and individual differences in general intellectual functioning, respectively. Model 3 included age and GIA scores at the student level and the linguistic demand of test directions and its interaction with age at the test level as fixed-effect predictors. The interaction between age and the linguistic demand of test directions was included to account for a potentially greater effect on younger students than on older students. This effect assumes that older students are typically less likely to have difficulty understanding the test directions compared to younger students. Models 4 and 5 included students’ oral expression and listening comprehension scores from the WJ IV OL as additional predictors, respectively. Finally, Model 6 included all the predictors (i.e., age, GIA, linguistic demand of test directions, and its interaction with age, oral expression, and listening comprehension) to predict the *W* scores. The mixed-effects models were estimated using the *lme4* package (Bates et al. 2015) in R (R Core Team 2021). The models were evaluated in terms of the statistical significance of the fixed-effect predictors, as well as the change in the amount of variance explained by the models.

## 3. Results

**Figure 1:**
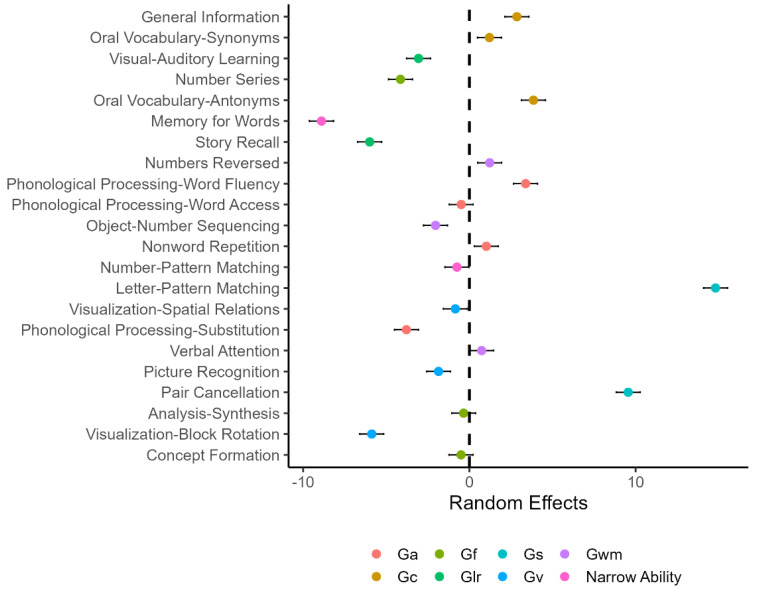
Estimated random effects and 95% confidence intervals for the tests in Model 1 (note: The dashed line represents the overall average score. The subtests on the *y*-axis are sorted in descending order by linguistic demand of the test directions).

The results of the mixed-effects models are summarized in Table 2 and Table 3. The variance estimates for Model 1 indicated that there was considerable variation in the students’ scores at both levels 1 and 2. The random effect estimates in Figure 1 essentially represent the differences between the average scores for the tests and the average score across all tests (i.e., vertical, dashed line). In addition, the WJ IV COG tests on the *y*-axis are sorted in descending order by the linguistic demand of the test directions. Relative to the overall average score, the average score for Memory for Words was the smallest, whereas the average score for Letter-Pattern Matching was the highest. Figure 1 shows that although the Gc tests (i.e., Comprehension-Knowledge) seem to have higher linguistic demand than the remaining tests, there is no systematic pattern in terms of the relationship between the average scores and the linguistic demand of their test directions. Figure 1 also indicates that the WJ IV COG tests differ regarding students’ performance on the tests. Thus, test- and student-level predictors can be used to further explain the variation among the tests.

The first model with fixed-effect predictors was Model 2. Table 2 shows that both the GIA scores and age were significant, positive predictors of student performance on individual cognitive tests. This result was anticipated given that all the abilities measured by the WJ IV COG are expected to continue to develop at an accelerated rate within the age range of the sample used for this study (see McGrew et al. 2014, p. 137). Similarly, the GIA score is expected to have a positive relationship with individual cognitive tests because it is the composite of the theoretical constructs (i.e., first-order factors) underlying the individual tests in the WJ IV COG. The GIA score is interpreted as a robust measure of statistical or psychometric *g*.

It should be noted that there are renewed debates regarding what intelligence test global composite scores (e.g., GIA, Wechsler Full-Scale IQ) represent. Briefly, the finding of a statistical or psychometric *g* factor is one of the most robust findings over the last 100+ years in psychology (Wasserman 2019). However, recent statistical and theoretical advances using network science methods (viz., psychometric network analysis; PNA) have suggested that psychometric *g* is only a necessary mathematical convenience and a statistical abstraction. The reification of the *g* factor in psychometrics is due, in large part, to the conflation of psychometric and theoretical *g* and has contributed to the theory crises in intelligence research (Fried 2020). Researchers using contemporary cognitive theories of intelligence (e.g., dynamic mutualism; process overlap theory; wired intelligence) have shown valid alternative non-latent trait common cause factor explanations of the positive manifold of intelligence tests. Furthermore, the previously mentioned period of 100+ years of research regarding general intelligence has also robustly demonstrated that there is yet no known biological or cognitive process theoretical basis of psychometric *g* (Barbey 2018; Detterman et al. 2016; Kovacs and Conway 2019; van der Maas et al. 2019; Protzko and Colom 2021). Therefore, in this paper, the GIA score is interpreted to reflect an emergent property that is a pragmatic statistical proxy for psychometric *g*, and not a theoretical individual differences latent trait characteristic of people. This conceptualization of the GIA score is similar to the emergent property of an engine’s horsepower: an emergent property index that summarizes the efficiency of the complex interaction of engine components (i.e., interacting brain networks), in the absence of a “horsepower” component or factor (i.e., theoretical or psychological *g*). Given that this debate is still unresolved after 100+ years of research, the GIA cluster was left in the analysis to acknowledge the possibility that theoretical or psychological *g* may exist and to recognize the strong pragmatic predictive powers of such a general proxy. Leaving GIA out of the analysis, which would reflect a strong “there is no *g*” position (McGrew et al. 2022), reflects the authors’ recognition that many intelligence scholars still maintain a belief in the existence of an elusive underlying biological brain-based common cause mechanism. More importantly, the inclusion of GIA recognizes the overwhelming evidence of the robust pragmatic predictive power of psychometric *g*. As such, the inclusion of age and the GIA scores in the model allowed us to control for their effects when including additional variables. The inclusion of age and the GIA scores also explained a large amount of variance at the student level (level 1), reducing the level-1 unexplained variance from 573.39 to 265.72 (i.e., 46% reduction).

When the variable *test directions* and its interaction with age were added to the model (Model 3), the variance estimate for level 2 (i.e., tests) remained relatively unchanged. This finding was mainly because *test directions* was not a significant predictor of the variation in the subtest scores. An interesting finding was that despite the variable *test directions* not being a significant predictor of cognitive test performance, its interaction with age was a statistically significant, negative predictor of cognitive test performance. This finding indicates that the impact of *test directions* was larger for younger students who are expected to have lower language proficiency compared with older students. This trend—*test directions* not being a significant predictor of individual cognitive test performance, but its interaction with age being a significant predictor of individual cognitive test performance—continued as the two additional variables were added to the model (see Models 4, 5, and 6 in Table 2). Models 4 and 5 showed that both *oral expression* and *listening comprehension* were statistically significant, positive predictors of individual cognitive test performance, after controlling for the effects of age and general intelligence.

Another important finding was that when both *oral expression* and *listening comprehension* were included in the model (Model 6), the two predictors remained statistically significant. The standardized beta coefficients produced from models 4 and 5 suggest that oral expression and listening comprehension were stronger predictors of cognitive test performance than students’ age, but relatively weaker predictors compared with the GIA score (see Table 3). When these four predictors (age, GIA, oral expression, and listening comprehension) were used together in the final model (Model 6), the GIA remains the strongest predictor, followed by listening comprehension (receptive language ability), oral expression (expressive language ability), and age, based on the standardized coefficients. The standardized beta coefficients also provided additional information regarding the negligible contribution of test directions within the models.

## 4. Discussion

The results of this study represent an integration of multiple pieces of empirical research completed over several years and across numerous studies. When examined in isolation, it appeared that examinee characteristics and test characteristics (e.g., test directions) *both* played meaningful roles in the administration and interpretation of cognitive tests for students. However, the integration of these potential influences into a single model has led to a surprising finding: the influence of examinee characteristics appears to eliminate the contribution of this test characteristic (i.e., the linguistic demand of test directions) on test performance. In addition, the influence of language ability, particularly receptive language ability, is more influential than age on cognitive test performance. This last point highlights the importance of considering language abilities when assessing students’ cognitive abilities.

### 4.1. Examinee versus Test Characteristics

There appears to be increasing evidence that previous claims related to test characteristics (e.g., Cormier et al. 2011) no longer apply to contemporary tests of cognitive abilities. For example, the observed lack of a relationship between test directions and performance on the WJ IV in our study appears to draw a parallel with comments made by Cormier, Wang, and Kennedy, as they observed a “reduction in the relative verbosity of the test directions” when comparing the most recent version of the Wechsler Intelligence Scale for Children (WISC; Wechsler 2014) to the previous version (Wechsler 2003). These findings, in addition to the generation of clear guidelines for test development (e.g., AERA et al. 2014), both support the notion that large-scale, standardized measures include greater evidence of validity for the diverse population from which they are normed. This, in turn, likely contributes to increased fairness for the students that are assessed using these tests.

Despite the advances in test development, considerable challenges in assessing EL students remain for psychologists. One such challenge is assessing the cognitive abilities of the growing number of students who are considered ELs; limited English proficiency can lead to linguistically biased test results, which would lead to a misrepresentation of the examinee’s true cognitive abilities. To eliminate this potential source of bias, psychologists testing EL students could consider examinee characteristics *before* administering a standardized measure of cognitive ability. This idea is not new. More than a decade ago, Flanagan et al. (2007) noted the critical need for psychologists to collect information regarding students’ level of English proficiency, and the level of English required for the student to be able to comprehend test directions, formulate and communicate responses, or otherwise use their English language abilities within the testing process. Nonetheless, the results of our study provide an *empirical basis* in support of this broad recommendation.

### 4.2. Assessing English Language Abilities

The primary reason for assessing an examinee’s English language skills is to determine if the examinee has receptive and expressive language skills that are comparable to the measure’s normative sample. However, relying on one’s clinical judgment when assessing an examinee’s expressive and receptive language abilities is not likely to lead to positive outcomes. If practitioners only rely on their own judgment to determine the examinee’s receptive and expressive language abilities, this could lead to either an under- or over-estimation of these abilities. An under-estimation could occur if the examiner deviates from the standardized administration because they do not believe that the examinee has understood the directions. Thus, the linguistic demand of the actual, standardized test directions is potentially reduced. An over-estimation may occur if the examiner disregards the influence of the examinee’s language abilities during testing and the results are interpreted the same for all examinees, regardless of their language abilities. In either case, an examiner who relies on their own judgment introduces unnecessary error into the assessment process. Therefore, especially in the context of testing EL students, practitioners should collect *data* on the receptive and expressive language abilities of examinees, so they can more accurately and reliably consider the potential influence of these variables on test performance.

Testing both expressive and receptive language abilities is critical for several other reasons. First, the results of the current study suggest that both make unique contributions to cognitive test performance. Second, a student’s receptive and expressive language abilities are not always at the same level of proficiency. Moreover, although a student’s conversational level of English language proficiency could be perceived to be relatively consistent with their peers’, their level of academic language proficiency may not be sufficient to fully benefit from classroom instruction or understand test directions to the same extent of a native English language speaker (Cummins 2008).

Some practitioners may have concerns regarding the additional testing time required to administer, score, and interpret performance on language ability tests. Flanagan et al. (2013) addressed this concern well, as they explained:

Irrespective of whether test scores ultimately prove to have utility or not, practitioners must endeavor to ascertain the extent to which the validity of any obtained test scores may have been compromised prior to and before any interpretation is offered or any meaning assigned to them.

(p. 309)

Therefore, not only would this process be consistent with the aforementioned standards, but it would also lead to recommendations that are better informed and tailored to individual examinee characteristics.

### 4.3. Limitations and Future Research

This study was the first to integrate multiple sources of influence on test performance using a large, representative sample of the United States school-age population. However, the study was not without limitations, some of which may inform future efforts to continue with this line of inquiry. First, although the large, representative sample used in this study contained a wide range of language ability levels, it did not contain many EL students. Continuing to investigate the extent to which examinee characteristics matter, particularly within a well-defined sub-population of EL students, would better inform best practices with respect to the assessment of EL students.

The influence of examiner characteristics was noted as one of the three potential contributors to test performance. However, only examinee characteristics and test characteristics were included in the models produced for this study. Although examiner characteristics likely precede the psychoeducational assessment process with respect to assessing one’s ability to engage in culturally sensitive practices and mastering the various aspects of test administration, scoring, and interpretation, their potential influence on test performance for EL students could be the focus of future research.

## Figures and Tables

**Table 1:** List of WJ IV Tests of Cognitive Abilities and WJ IV Tests of Oral Language.

WJ IV Tests of Cognitive Abilities—Subtest	WJ IV Tests of Oral Language (English Tests Only)
Oral Vocabulary—Synonyms	Picture Vocabulary ^2^
Oral Vocabulary—Antonyms	Oral Comprehension ^3^
Number Series	Segmentation
Verbal Attention	Rapid Picture Naming
Letter-Pattern Matching	Sentence Repetition ^2^
Phonological Processing—Word Access	Understanding Directions ^3^
Phonological Processing—Word Fluency	Sound Blending
Phonological Processing—Substitution	Retrieval Fluency
Story Recall	Sound Awareness
Visualization—Spatial Relations	
Visualization—Block Rotation	
General Information ^1^	
Concept Formation	
Numbers Reversed	
Number-Pattern Matching	
Nonword Repetition	
Visual-Auditory Learning	
Picture Recognition	
Analysis-Synthesis	
Object-Number Sequencing	
Pair Cancellation	
Memory for Words	

^1^ The General Information test score is produced from the General Information—What and General Information—Where subtests. The subtests are not listed in the table because the analysis was completed at the test level for General Information; ^2^ The Picture Vocabulary and Sentence Repetition tests produce the Oral Expression cluster score; ^3^ The Oral Comprehension and Understanding Directions tests produce the Listening Comprehension cluster score.

**Table 2:** A Summary of the Results from the Mixed-Effects Models.

		Variances		Fixed Effects		
Model	Predictors	Level 1	Level 2	*β*	*t*	*p*
1	Intercept	573.39	25.12	502.10	469	<0.001
2	Intercept	265.72	25.14	45.67	19.88	<0.001
	Age			0.02	9.83	<0.001
	GIA			0.90	203.84	<0.001
3	Intercept	265.68	25.13	45.59	19.79	<0.001
	Age			0.02	10.15	<0.001
	GIA			0.90	203.85	<0.001
	Test directions			0.53	0.47	0.643
	Test directions × Age			−0.01	−3.61	<0.001
4	Intercept	265.28	25.13	41.56	17.86	<0.001
	Age			0.01	8.41	<0.001
	GIA			0.86	145.88	<0.001
	Test directions			0.53	0.47	0.643
	Test directions × Age			−0.01	−3.61	<0.001
	Oral expression			0.05	11.79	<0.001
5	Intercept	265.24	25.13	33.46	13.37	<0.001
	Age			0.02	9.16	<0.001
	GIA			0.84	128.74	<0.001
	Test directions			0.53	0.47	0.643
	Test directions × Age			−0.01	−3.61	<0.001
	Listening comprehension			0.08	12.38	<0.001
6	Intercept	265.12	25.13	34.53	13.77	<0.001
	Age			0.01	8.34	<0.001
	GIA			0.83	123.01	<0.001
	Test directions			0.53	0.47	0.643
	Test directions × Age			−0.01	−3.61	<0.001
	Oral expression			0.03	6.52	<0.001
	Listening comprehension			0.06	7.53	<0.001

Note: GIA = General Intellectual Ability. In this paper GIA is conceptualized as a manifest indicator that represents statistical or *psychometric g*, and not a *theoretical* or *psychological g* latent brain-based biological or cognitive process dimension (see Fried 2020; McGrew et al. 2022).

**Table 3:** Standardized Beta Coefficients for Models 4, 5, and 6.

	Predictors					
Model	Age	*g*	Oral Expression	Listening Comprehension	Test Directions	Test Directions × Age
4	**0.029**	**0.656**	**0.047**		0.021	**−0.026**
5	**0.031**	**0.645**		**0.055**	0.021	**−0.026**
6	**0.029**	**0.637**	**0.030**	**0.039**	0.021	**−0.026**

Note: Bold values indicate significant predictors in the models.

## Data Availability

The data presented in this study are not publicly available because they are confidential and proprietary (i.e., owned by the WJ IV publisher). Requests to access the data should be directed to Riverside Insights.
